# Three New Species of *Cyphellophora* (Chaetothyriales) Associated with Sooty Blotch and Flyspeck

**DOI:** 10.1371/journal.pone.0136857

**Published:** 2015-09-23

**Authors:** Liu Gao, Yongqiang Ma, Wanyu Zhao, Zhuoya Wei, Mark L. Gleason, Hongcai Chen, Lu Hao, Guangyu Sun, Rong Zhang

**Affiliations:** 1 Department of State Key Laboratory of Crop Stress Biology in Arid Areas and College of Plant Protection, Northwest A&F University, Yangling, Shaanxi Province, China; 2 Institute of Plant Protection, Qinghai Academy of Agricultural and Forestry Sciences, Xining, Qinghai Province, China; 3 Department of Plant Pathology and Microbiology, Iowa State University, Ames, Iowa, United States of America; The University of Hong Kong, HONG KONG

## Abstract

The genus *Cyphellophora* includes human- and plant-related species from mammal skin and nails, plant materials, and food. On the basis of analysis of ITS, LSU, TUB2 and RPB1 data and morphological characters, three new species, *Cyphellophora phyllostachysdis*, *C*. *artocarpi* and *C*. *musae*, associated with sooty blotch and flyspeck disease, were added to this genus. The 2D structure of ITS1 and ITS2 confirmed this taxonomic status. Pathogenicity tests on apple fruit indicated that *C*. *artocarpi* could be a sooty blotch and flyspeck pathogen of apple.

## Introduction

The genus *Cyphellophora* de Vries (Cyphellophoraceae, Chaetothyriales) was set up in 1962 with *C*. *laciniata* as the type species. It was characterized by producing septate conidia from intercalary, terminal or lateral phialides bearing flaring, thin or conspicuous collarettes [1]. Subsequently, *C*. *pluriseptata* [2], *C*. *vermispora* [3], *C*. *taiwanensis* [4], *C*. *indica*, *C*. *guyanesis*, *C*. *suttonii*, *C*. *fusarioides* [5], and *C*. *pauciseptata* [6] were added to this genus. The genus *Phialophora* Medlar (Chaetothyriales) was established in 1915 [7]. The morphological characters of *Phialophora* resemble those of *Cyphellophora* in possessing phialides and collarettes on conidiogenous cells, but *Phialophora* produces single-celled conidia whereas *Cyphellophora* produces multiseptate conidia. Phylogenetic analysis revealed that *Phialophora* was polyphyletic; some species were outside of the lineage containing *P*. *verrucosa* (type species) and situated in a sister clade with *C*. *laciniata* [8]. Feng et al. expanded this clade to some species of *Phialophora* and most species of *Cyphellophora* by phylogenetic analysis of large subunit nuclear ribosomal DNA (LSU), noting that many species in this clade cause superficial infections in humans [6]. Réblová et al. [9] accommodated this unique clade to a new family, Cyphellophoraceae, under Chaetothyriales, and all the species in this clade were transferred to genus *Cyphellophora* on the basis of multi-locus gene analysis and secondary (2D) structure of ITS analysis. Morphology of *Cyphellophora* G.A. de Vries emend. Réblová & Unter. was expanded to encompass species with both septate and nonseptate conidia.

Ecological niches of *Cyphellophora* species are widespread. The type species, *C*. *laciniata*, along with *C*. *europaea* and *C*. *pluriseptata*, was isolated from human skin and nails, resulting in clinical symptoms [1, 2, 10]. In contrast, *Cyphellophora guyanensis*, *C*. *oxyspora*, *C*. *olivacea* and *C*. *sessilis* were isolated from plant materials or non-biological substrates. [5, 11, 12]. *Cyphellophora sessilis* (= *Phialophora sessilis*) was reported to cause sooty blotch and flyspeck, an economically damaging disease of certain fruit crops, as well as epiphytic colonies on bamboo [13–16].

Sooty blotch and flyspeck (SBFS) is a late-season fungal disease that occurs worldwide in areas with moist climates. The SBFS fungi colonize and blemish the epicuticular wax layer of the fruit of apple, pear, orange, persimmon, banana and several other cultivated tree and vine crops [17]. Although SBFS fungi are epiphytes and do not cause yield losses [18–20], they blemish the appearance of fresh fruit, reducing its sale value, and limit the growth of organic fruit production [19, 21, 22].

Fungi in the SBFS complex are highly diverse, comprising more than 80 putative and named species based on genetic and morphological evidence [14, 17, 21, 23–28]. Most of these species are grouped within the genera *Dissoconium*, *Ramichloridium*, *Peltaster*, *Microcyclosporella*, and *Zygophiala* (Capnodiales, Dothideomycetes). Until now, *Cyphellophora sessilis* has been the only *Cyphellophora* species known to cause SBFS [13, 29].

During a survey of SBFS fungi in southern China, *Cyphellophora* isolates were obtained from plant hosts. The objective of this study was to identify these isolates based on morphological characteristics, phylogenetic analysis of the internal transcribed spacer region (ITS), the partial beta tubulin gene (TUB2), the nuclear large subunit rDNA gene (LSU) and RNA polymerase II largest subunit (RPB1) and the 2D structure of ITS1 and ITS2. The pathway of melanin synthesis was also explored in this study.

## Materials and Methods

### Isolates

Five *Cyphellophora* isolates were obtained in this study. Two were obtained from colonies exhibiting SBFS colony morphology on bamboo species (*Phyllostachys heterocycla* [Carr.] Mitford and *Sinobambusa tootsik* [Sieb.] Makino) at the Botanical Garden of Haikou City, Hainan Province, and Maoming City in Guangdong Province, China, respectively. Two additional isolates were obtained from cuticles of Japanese banana (*Musa basjoo* Sieb. & Zucc.) fruit in Guangzhou and Zhanjiang City, Guangdong, and the final isolate came from a branch of jackfruit (*Artocarpus heterophyllus* Lam.) in Haikou City, Hainan Province. No specific permissions were required for sampling these locations as the plant are not rare or endangered species. The field studies did not involve endangered or protected species. Sclerotium-like bodies of colonies were transferred directly from host plants to potato dextrose agar (PDA) slants in a sterile environment, and incubated at 25°C for 1 month in darkness [30]. Representative isolates were deposited in the China General Microbiological Culture Collection Center (CGMCC) (Beijing, China) and dried cultures were deposited at the Herbarium Mycologicum Academiae Sinicae (HMAS) (Beijing, China). Host tissues exhibiting flyspeck colonies were excised and pressed between paper towels until dry, preserved in glycerol at -80°C, and deposited in the collection of the Fungal Laboratory of Northwest A&F University. Nomenclatural novelties and descriptions were deposited in MycoBank (www.MycoBank.org) [31]. The isolate coda, locations, hosts and GenBank numbers used in the study are shown in S1 Table

### Morphology of isolates

Hyphal tips of each isolate were transferred to OA (oatmeal agar) plates and descriptions of colony morphology were made after incubation for 4 weeks in darkness at 24°C. In order to measure and observe fungal structures, each isolate of *Cyphellophora* was allowed to grow onto an adjacent, sterile cover slip that had been partially inserted into the agar surface at a 60° angle [30]. Wherever possible, 30 measurements were made of structures mounted in lactic acid, with the extremes of measurements given in parentheses. For conidial measurements, the 95% percentiles are presented and extremes given in brackets [23].

### DNA extraction, PCR, and sequencing

Amplification of isolates was performed at Northwest A&F University. Genomic DNA was extracted from single-conidium isolates that had grown on PDA plates at 25°C in darkness for 4 to 6 wk. DNA was extracted from the mycelium according to the protocol of Li et al. [24]. Four genes were amplified: the internal transcribed spacer region (ITS), the partial β-tubulin gene (TUB2), the nuclear large subunit rDNA gene (LSU) and the DNA dependent RNA polymerase II largest subunit (RPB1). Primers used for amplification and sequencing were ITS1-F/ITS4 [32] for ITS, LR5/LROR [33, 34] for LSU, Bt2a/Bt2b [35], T2 [36] used as alternative forward primer for amplifying TUB2 and RPB1-Af/RPB1-Cr [37] for RPB1, respectively. Amplification reactions consisting of 1 unit of Taq polymerase, 1 × PCR buffer, 2 mM MgCl_2_, 0.2 mM of each dNTP, 0.4 μM of each primer, and 2 μL of template DNA were made up to a total volume of 25 μL with sterile water. Reactions were performed on a Bio-Rad PCR System PTC-200TM. Cycling conditions were an initial denaturation at 94°C for 95 s followed by 35 cycles of denaturation at 94°C for 35 s, annealing at 52°C for ITS, 49°C for LSU for 60 s, extension at 72°C for 2 min, and a final 10-min extension step at 72°C. The cycling parameters for the TUB2 region consisted of a 3-min denaturing step at 94°C followed by 34 cycles at 94°C for 45 s, annealing at 52°C for 1 min, 72°C for 1 min and a final cycle of 10 min at 72°C. PCR amplifications for RPB1 were performed at 5 cycles of 30 s denaturation at 94°C, followed by primer annealing for 30 s at 55°C, and extension for 1 min at 72°C; followed by 5 cycles with an annealing temperature of 53°C and 30 cycles at 51°C, then an extension for the final 10 min at 72°C. The resulting PCR products were cloned into DH5-α competent cell with the pMD18-T Vector System cloning kits (Takara Biotechnology [Dalian] Co., Ltd.) following the protocols. Purifying and automated sequencing of the PCR products was performed at Organism Technology, Shanghai, China with forward and reverse primers.

### Sequence alignment and phylogenetic analysis

Phylogenetic relationships were based on the analysis of ITS gene and ITS-LSU-TUB-RPB1 matrix sequences. All sequences generated in this study, along with other *Cyphellophora* spp. and outgroup sequences downloaded from NCBI’s GenBank sequence database (S1 Table), were concatenated by FASTA alignment and imported into BioEdit [38] to be compared and analyzed. *Cladophialophora immunda* isolate CBS 834.96 was used to root the ITS and four-locus phylogenetic trees. Preliminary alignments of the multiple sequences were conducted using CLUSTAL X [39], with manual adjustment using BioEdit for visual improvement where necessary.

To estimate relationships within the genus *Cyphellophora*, maximum parsimony (MP) and Bayesian inference (BI) analysis were used. MrModeltest v. 2.2 [40] was used to determine the best nucleotide substitution model. Maximum parsimony (MP) analysis was performed with PAUP v.4.0b10 [41]. Heuristic searches were conducted with 1000 random sequence additions and tree bisection-reconnection (TBR) branch swapping algorithms, collapsing zero-length branches, and saving all minimal length trees [42]. All characters were unordered and of equal weight and gaps were treated as missing data. Branches of zero length were collapsed and all multiple, equally parsimonious trees were saved. To assess the robustness of clades and internal branches, a strict consensus of the most parsimonious trees was generated and a bootstrap analysis of 1,000 replications was performed. Bayesian inference (BI) analysis was conducted in a likelihood framework on the same aligned dataset. Phylogenetic analyses were performed with Mrbayes 3.2.2 [43] applying a general time-reversible (GTR) substitution model with gamma (G) and proportion of invariable site (I) parameters to accommodate variable rates across sites. The Markov Chain Monte Carlo (MCMC) analysis of four chains with two runs started from random tree topology and lasted 5,000,000 generations. Trees were saved each 1000 generations. The first 12,500 trees, which represented the burn-in phase of the analysis, were discarded. A 50% majority rule consensus tree and posterior probabilities (PP) for each split was calculated after excluding the first 1250 sampled trees. The remaining trees were used for calculating posterior probabilities (PP) of recovered branches in the 50% majority rule consensus tree.

**Fig 1:**
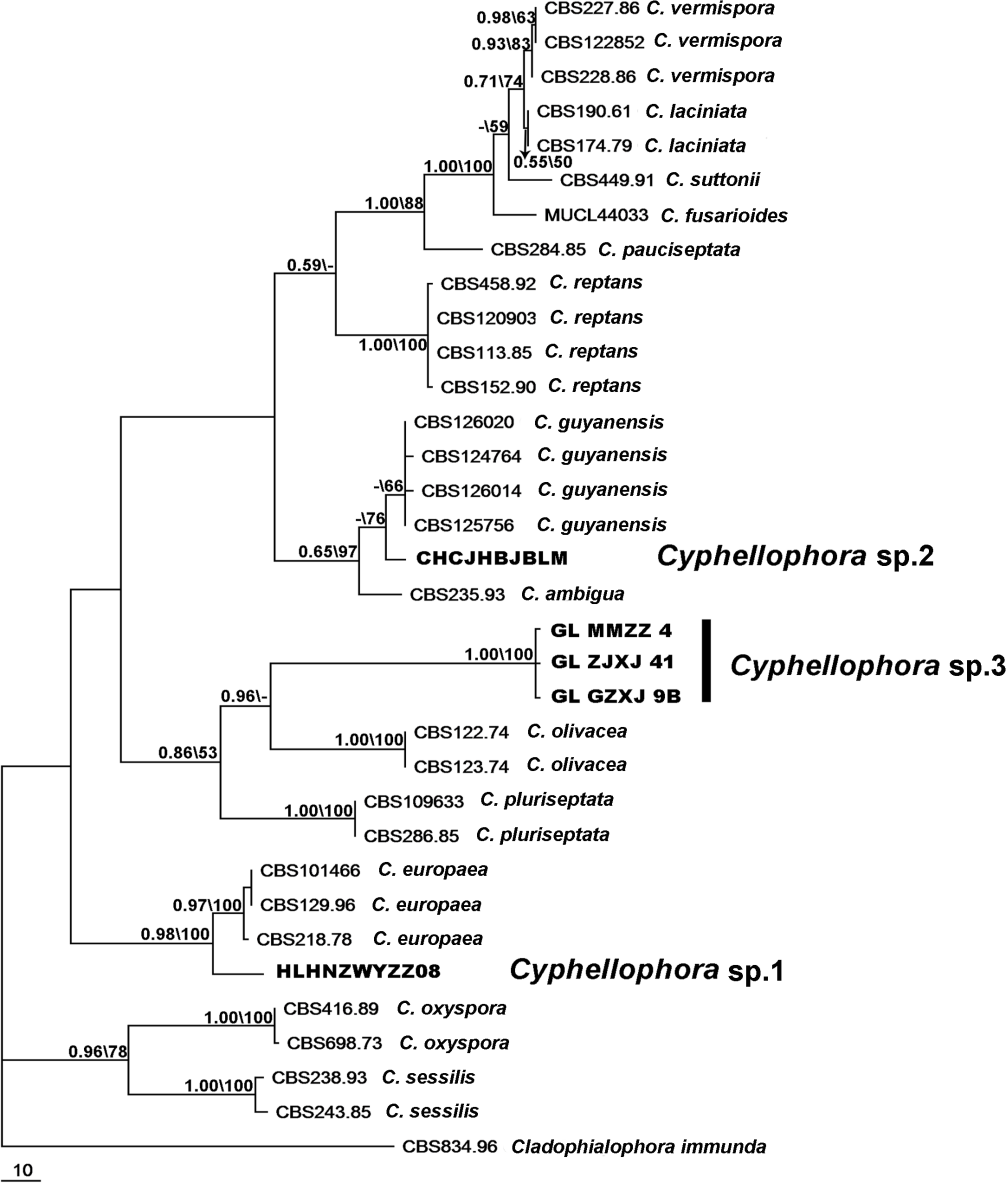
ITS phylogeny of *Cyphellophora*. One of 100 equally parsimonious trees determined from maximum parsimony analysis of ITS sequences. The first number shown at each node is the Bayesian posterior probability (BPP ≥ 0.5) and the second number represents maximum parsimony bootstrap proportion (MP-BS ≥ 50%). The tree is rooted to *Cladophialophora immunda* isolate CBS 834.96; new sequences generated in this study are printed in bold.

Bayesian posterior probabilities (BPP) ≥ 0.8 and bootstrap values ≥ 70% were considered significant. Bayesian posterior probabilities (BPP) > 0.5 and MP bootstrap support (BS) > 50% are given in Fig 1 at the first and second positions above or below the branches. Alignments and representative trees (Fig 1) were deposited in TreeBASE (http://purl.org/phylo/treebase/phylows/study/TB2:S16945).

### Prediction of 2D structure models of ITS1 and ITS2

Consensus 2D structure models for the ITS1 and ITS2 were built using the Mfold program [44] which depends on thermodynamic methods. The ITS2 Database III [45] with available structural models was also used to build consensus 2D structures. The predicted 2D RNA structures were obtained in a dot bracket notation and were visualized and drawn using VARNA: Visualization Applet for RNA program [46].

### Pathogenicity test on apple

From isolates used in morphological characterization, one isolate of each species was selected for pathogenicity tests on apple fruit in an incubator. Isolates HLHNZWYZZ-08, CHCJHBJBLM and GLZJXJ41, which were initially obtained from a twig of *Phyllostachys heterocycla*, a twig of *Artocarpus heterophyllus* and a fruit of *Musa basjoo*, respectively, were used for inoculation. Preparation of inoculum suspensions followed protocols of Batzer et al. [21]. Single-conidial isolates were grown on 2% potato-dextrose agar (PDA) for one month. Excess agar was cut away and the colony was transferred to three to four 1.5 ml plastic centrifuge tubes. Inoculum suspensions containing hyphae and conidia were blended with 600 ml sterile deionized water (SDW) and ground with a vortex oscillator (Model QL-901, Kylin-Bell Lab Instruments Company Limited, Haimen, Jiangsu Province, China) for 1 min. After that, they were refrigerated until use, which was within 2 h of preparation.

Inoculation in an incubator followed a field-based method described by Batzer et al. [21] and modified by Gao et al. [47]. Mature apples (cv. Gala) were used for inoculation. Apples were surface-sterilized with 70% ethanol and then allowed to dry for 2 min. For each isolate, five fruit were swabbed with inoculum suspension using sterilized Chinese brushes; on each apple, three inoculated areas, each 1 to 2 cm in diameter, were marked. Three control fruit were treated with distilled water instead of the inoculum suspensions. After inoculation, fruit were transferred to an incubator with 12 h fluorescent light at 28°C and 85% relative humidity (RH) alternated with 12 h darkness at 25°C with 100% RH for 6 wk, then examined for signs and photographed.

### Nomenclature

The electronic version of this article in Portable Document Format (PDF) in a work with an ISSN or ISBN will represent a published work according to the International Code of Nomenclature for algae, fungi, and plants, and hence the new names contained in the electronic publication of a PLOS ONE article are effectively published under that Code from the electronic edition alone, so there is no longer any need to provide printed copies.

In addition, new names contained in this work have been submitted to MycoBank from where they will be made available to the Global Names Index. The unique MycoBank number can be resolved and the associated information viewed through any standard web browser by appending the MycoBank number contained in this publication to the prefix http://www.mycobank.org/MB/. The online version of this work is archived and available from the following digital repositories: PubMed Central, LOCKSS.

## Results

### Phylogeny

For ITS alignment, 34 sequences with 623 characters were used. Of 100 identical topological MP trees produced by PAUP, one is shown in Fig 1; isolates generated in this study are shown in bold. The ITS tree was 613 steps in length with consistency index (CI) of 0.6558 and retention index (RI) of 0.8316. Average standard deviation of split frequencies from Bayesian analysis were below 0.01 after 5 000 000 generations. The tree produced by Bayesian phylogenetic analysis showed similar topology that agreed on clustering and branching patterns; the Bayesian posterior probabilities (BPP) are shown along with bootstrap support (BS) on the branches of the tree (Fig 1). According to ITS analysis, three isolates, GLMMZZ4, GLZJXJ41 and GLGZXJ9B, separated with *C*. *olivacea*, clustered as a new branch with high support (BS = 100; BPP = 1.0), and resolved as a unique clade. Another isolate obtained in this study, CHCJHBJBLM, differed from *C*. *guyanensis* with 76% bootstrap support. However, no Bayesian posterior probability (BPP) was given as the value was not strong enough. The third cluster (HLHNZWYZZ08) formed a new branch with high support (0.98 and 100% for BPP and BS, respectively). Twenty base pair differences separated this cluster from *C*. *europaea* strain CBS 101466.

**Fig 2:**
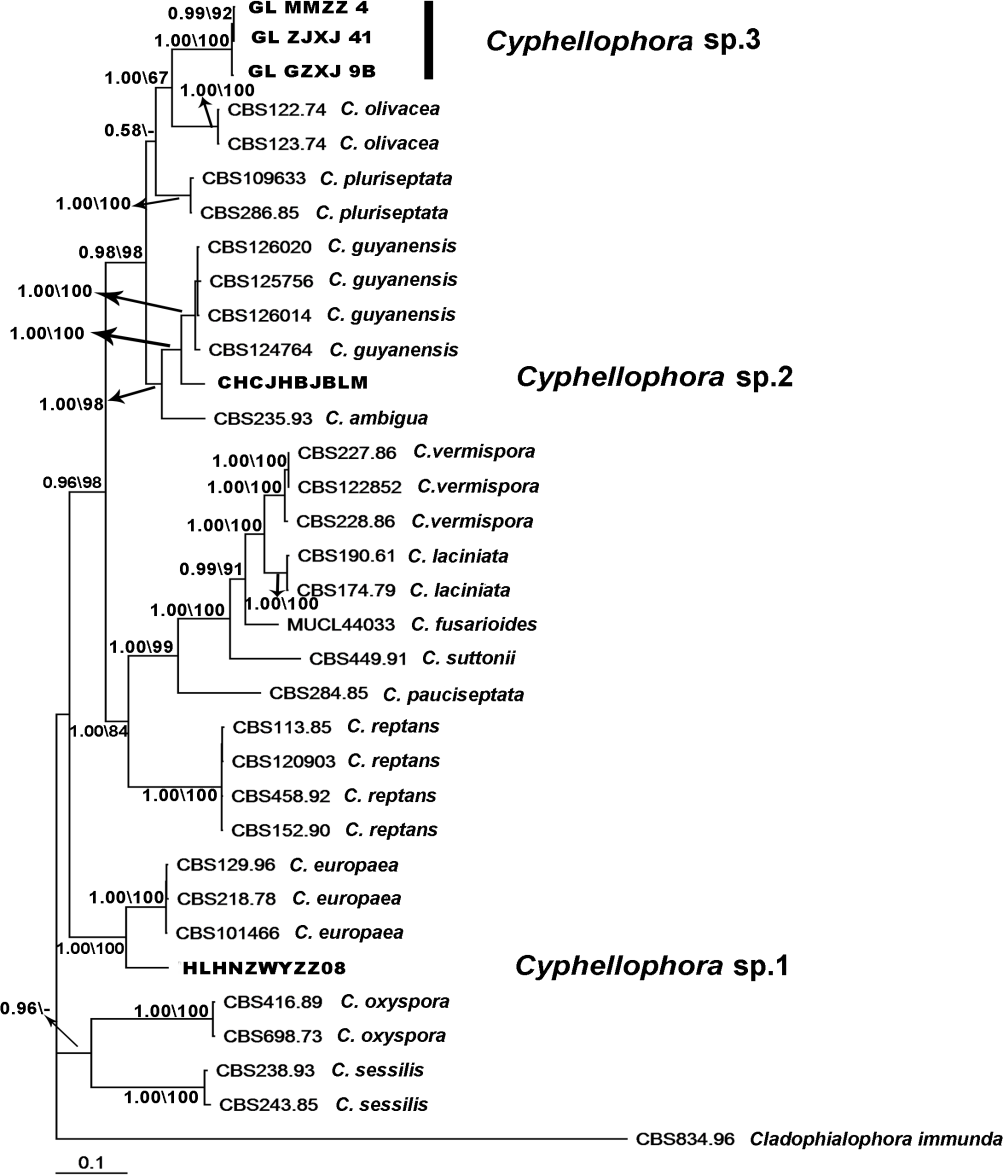
Bayesian tree based on combined data set of ITS, LSU, TUB and RPB1 sequences. Bayesian posterior probability ≥ 0.5 and maximum parsimony bootstrap support (MP-BS) ≥ 50% are shown above and below the branches in first and second positions, respectively. New sequences generated in this study are printed in bold. The RPB1 sequences of *C*. *olivacea*, *C*. *sessilis* and *Cladophialophora immunda* are missing.

The combined gene alignment of ITS, LSU, TUB and RPB1 included 2513 characters, of which 774 were parsimony-informative. The RPB1 sequences of *C*. *olivacea* and *C*. *sessilis* were missing as we did not obtain these sequences from GenBank. Eight identical topological MP trees were produced with 2721 steps in length; the consistency index (CI) was 0.5524 and the retention index (RI) was 0.7936. The tree obtained from Bayesian phylogenetic analysis shared a similar topology with MP trees and is shown in Fig 2 The four-locus dataset alignment generated a topology tree similar to those inferred in the ITS analysis, but support for branches in the combined phylogeny was generally stronger. The isolates obtained in this work were identified as three new species of *Cyphellophora* based on their morphology in culture, growth characteristics and DNA sequence (ITS, LSU, TUB and RPB1) phylogeny.

## 2D structure of ITS1 and ITS2

**Fig 3:**
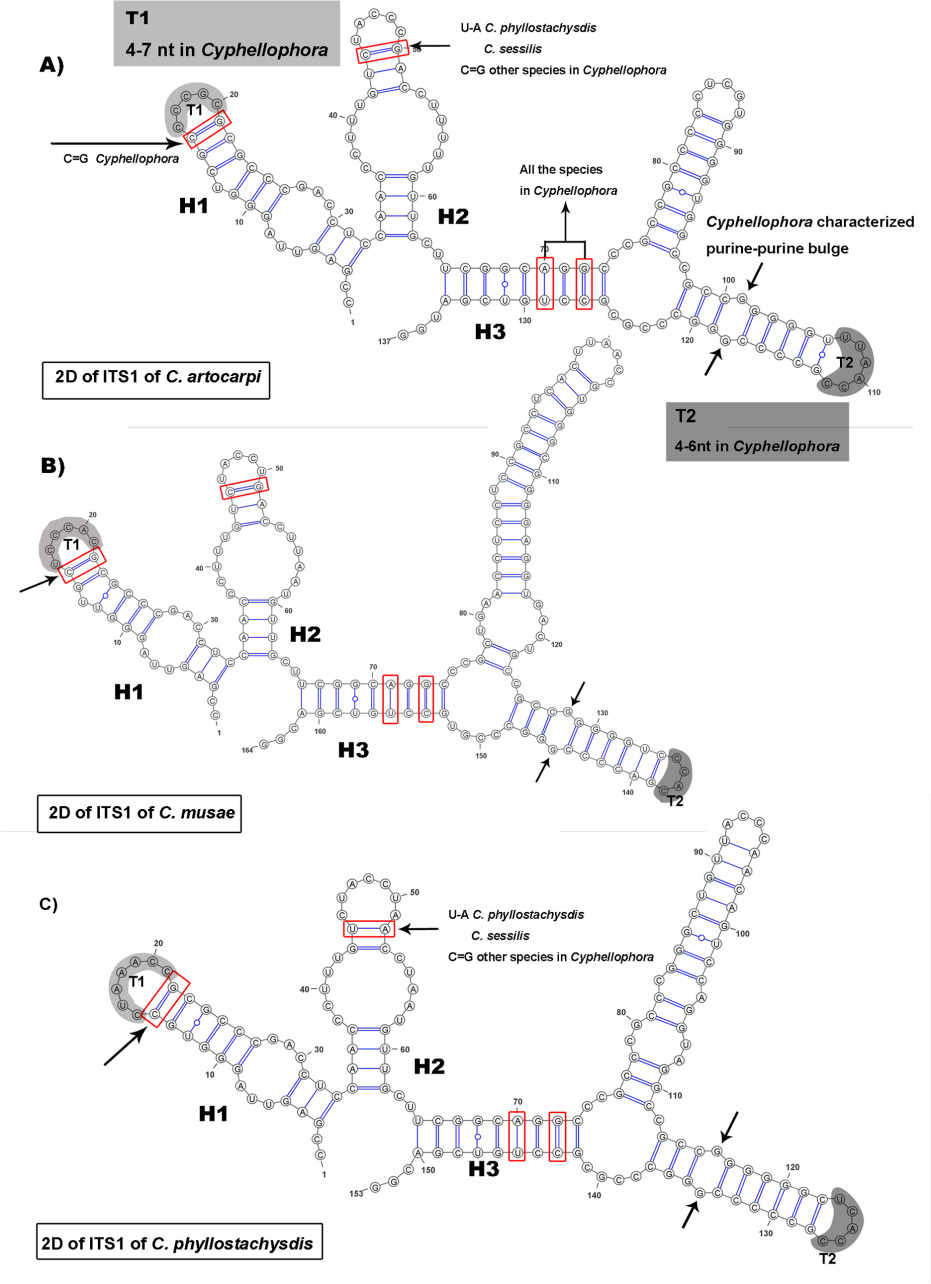
Predicted models of ITS1 rRNA secondary structure. Predicted models of A) *C*. *artocarpi*, B) *C*. *musae*, and C) *C*. *phyllostachysdis*. The structures contain three helices numbered H1–H3. The grey-colored parts of hairpin loops(T1, T2) are regions with variable numbers of nucleotides. The identity and difference of the structures among different *Cyphellophora* species are mapped on the 2D models.

**Fig 4:**
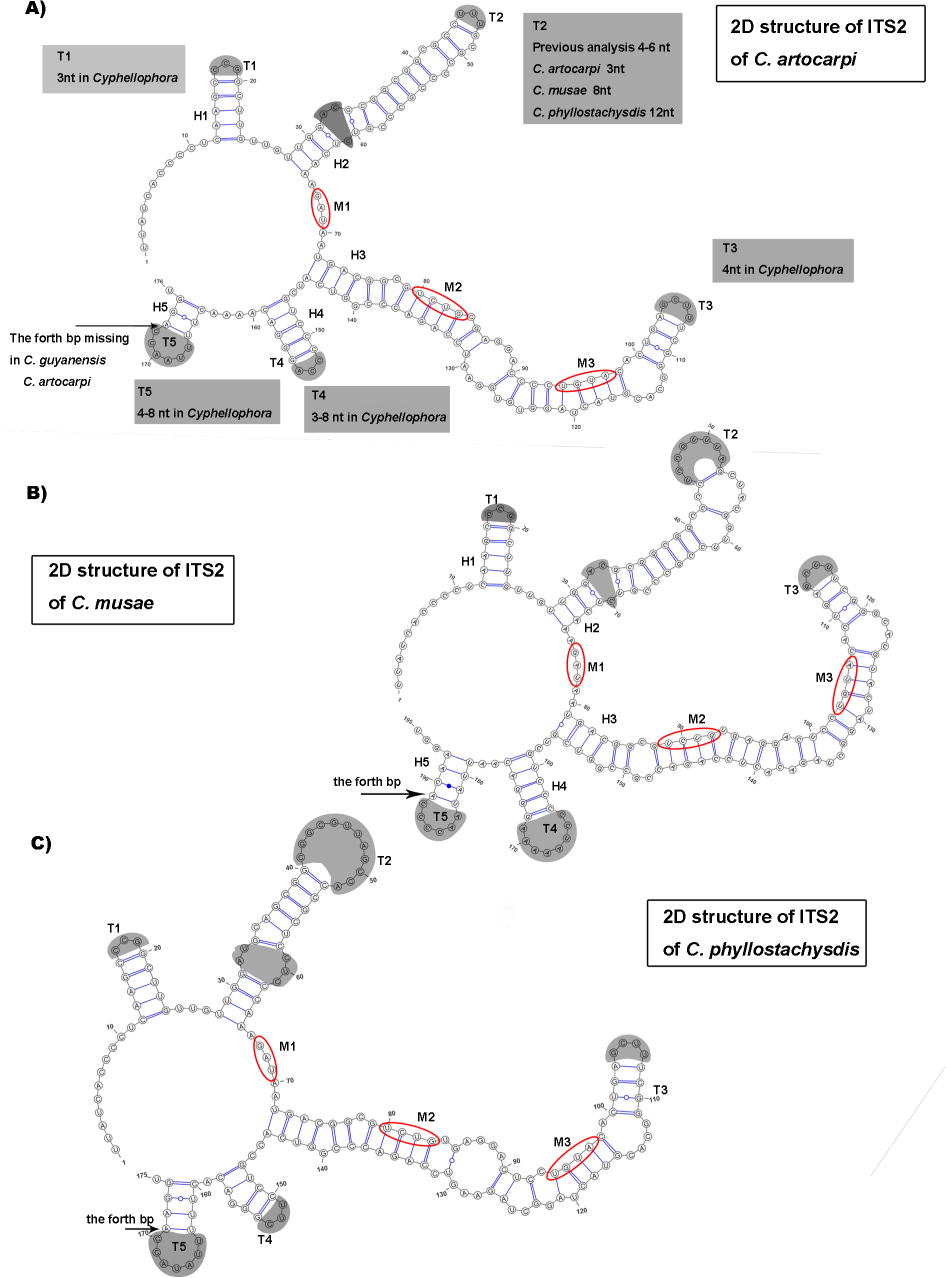
Prediction of ITS2 rRNA secondary structure models of *C*. *artocarpi*, *C*. *musae*, and *C*. *phyllostachysdis*. Predicted models of A) *C*. *artocarpi*, B) *C*. *musae*, and C). *C*. *phyllostachysdis*. Five helices in the 2D structures are numbered H1–H5. Five hairpin loops (T1–T5) are highlighted with grey.

The structures of ITS1 and ITS2 of *C*. *phyllostachysdis*, *C*. *artocarpi* and *C*. *musae* predicted by 2D analysis are shown in Figs 3 and 4, respectively. The non-homologous regions in ITS1 which were proposed by Réblová et al. [9] were removed.

The partial 2D structure of ITS1 of the three *Cyphellophora* species described in this study shared the same characters as that of *C*. *laciniata* [9]. It contained three helices (H1, H2 and H3) (Fig 3) separated by an adjacent single-stranded (junction) region. H1 was about 30-nt long and exhibited a symmetrical internal loop and a hairpin loop T1 of 4–7 nt in all *Cyphellophora* species. The C = G bp framed with red box existed in *Cyphellophora*. H2 was also approximately 30-nt long and included an internal loop and a hairpin loop. In most species of *Cyphellophra* (except *C*. *sessilis* and *C*. *phyllostachysdis*), the framed bp is C = G. The U-A nt exists in *C*. *sessilis* and *C*. *phyllostachysdis*, *Aphanophora*, *Camptophora* and most members of the Chaetothyriaceae. In other taxa these two nt do not pair and become part of the loop [9]. H3 is branched and it is the longest of the helices. The purine-purine pair indicated by the arrows only exists in Cyphellophoraceae.

The 2D structures of ITS2 folded into five helices (H1–H5) (Fig 4). The structures described in this study also share the common characters of Cyphellophoraceae [9]. H1 was about 13 nt and consisted of 5 bp and a hairpin loop T1 (3 nt in *Cyphellophora*). According to Réblová et al. [9], the bottom portion of H1 is highly variable among members of the Cyphellophoraceae. Our three species presented the same structure as that of *C*. *europaea*. H2 was longer than H1 (about 35–45 nt), and was composed of an asymmetrical loop and a hairpin loop T2. Previous description of the shaded asymmetrical loop in *Cyphellophora* contained no more than two nt [9], whereas those of *C*. *artocarpi* and *C*. *musae* had 3 nt and *C*. *phyllostachysdis* contained up to 5 bp. H3 was the longest helix and consists several internal loops, bulges and a hairpin loop T3. The fourth duplex H4 had 4–5 bp and a hairpin loop T4. The last duplex H5 was the shortest one. Most *Cyphellophora* species have 4 bp except *C*. *guyanensis* and *C*. *artocarpi*, which have only 3 bp. The three evolutionary motifs in ITS2 are also consistent with that of Cyphellophoraceae (M1: GA(G)U; M2: UCUG; M3: UGUA).

### Taxonomy

**Fig 5:**
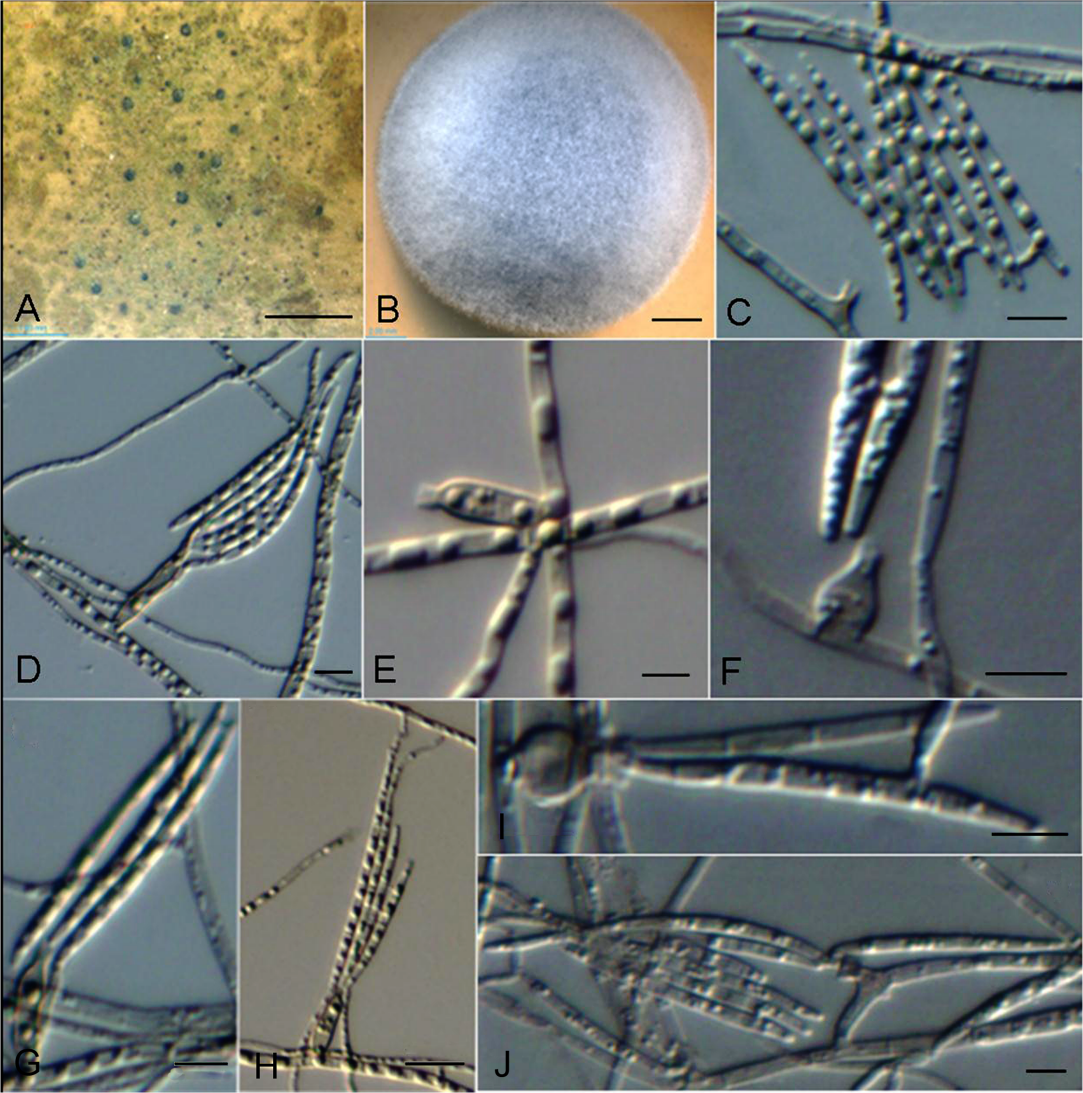
*Cyphellophora phyllostachysdis*. A, colonies on bamboo with close-up view. B, Colony on OA after 4 wk. C–J, Phialidic conidiogenous cell and conidia. Microscopic morphology on PDA: C, D, E, G, H; OA: F, I, J. *Scale bars*: A = 1mm, B = 2mm, C–G = 5μm, H = 10μm, I, J = 5μm.

***Cyphellophora phyllostachydis***. G. Y. Sun & Liu Gao, sp. nov. (Fig 5)

MycoBank MB811435.

Hyphae thin- to slightly thick-walled, hyaline to pale grayish, smooth, septate, ramose, aerial, rarely immersed, 1.0–1.5 μm wide. Conidiophores absent or rarely reduced to a short cell basal to the conidiogenous cells. Conidiogenesis enteroblastic, phialidic. Phialides ampulliform to short cylindrical, mostly arising at right angles from undifferentiated hyphae, rarely terminal, intercalary or lateral, sub-hyaline to pale olivaceous brown, thin-walled. Ampulliform-shape phialides 4(–5.5) × 4(–5) μm, short cylindrical (8–)15(–21) × 2–2.5(–3) μm (with a conspicuous, funnel-shaped collarette, 1.0 × 1.5 μm, sometimes lighter in color than the body of the phialides). Conidia produced from phialides of collarettes, sub-hyaline, smooth, nearly straight to more commonly falcate or curved, with slightly truncate basis and rounded apices, 0–3 septa, 25–30(–45) × 1.5–2 μm, sometimes agglomerating into groups. Chlamydospores and sclerotium absent.

Colonies on OA medium fast growing, attaining a diameter of 42 mm after four weeks at 25 C, with a woolly-velvety texture, mouse grey in the center discolouring progressively to pale grayish near the margin. Reverse pale brown. Regular margin, slightly raised center.

*Etymology*: Named after the host from which it was collected.

*Holotype*: CHINA. HAINAN: Haikou. From twig of *Phyllostachys heterocycla* (Carr.) Mitford, Oct 2010, L. Hao, HLHNZWYZZ-08 (Holotypus HMAS245769, ex-type culture CGMCC3.17495).

*Notes*: Morphologically, conidia shape is similar to *C*. *suttonii* (Ajello, A.A. Padhye & M. Payne) Decock (= *Pseudomicrodochium suttonii*) [5], which produces straight to falcate, acicular conidia in more or less sympodial order. The difference between these two species was that *C*. *suttonii* conidia were described as 3–8-septate, rather than 0–3 in our strain. The conidia of *C*. *phyllostachysdis* also resemble those of *C*. *guyanensis* in length and shape [5]; the latter was first isolated from a dead leaf of an undetermined angiosperm. However, number of septa per conidium (3–6 septa) exceeds that of *C*. *phyllostachysdis*.

**Fig 6:**
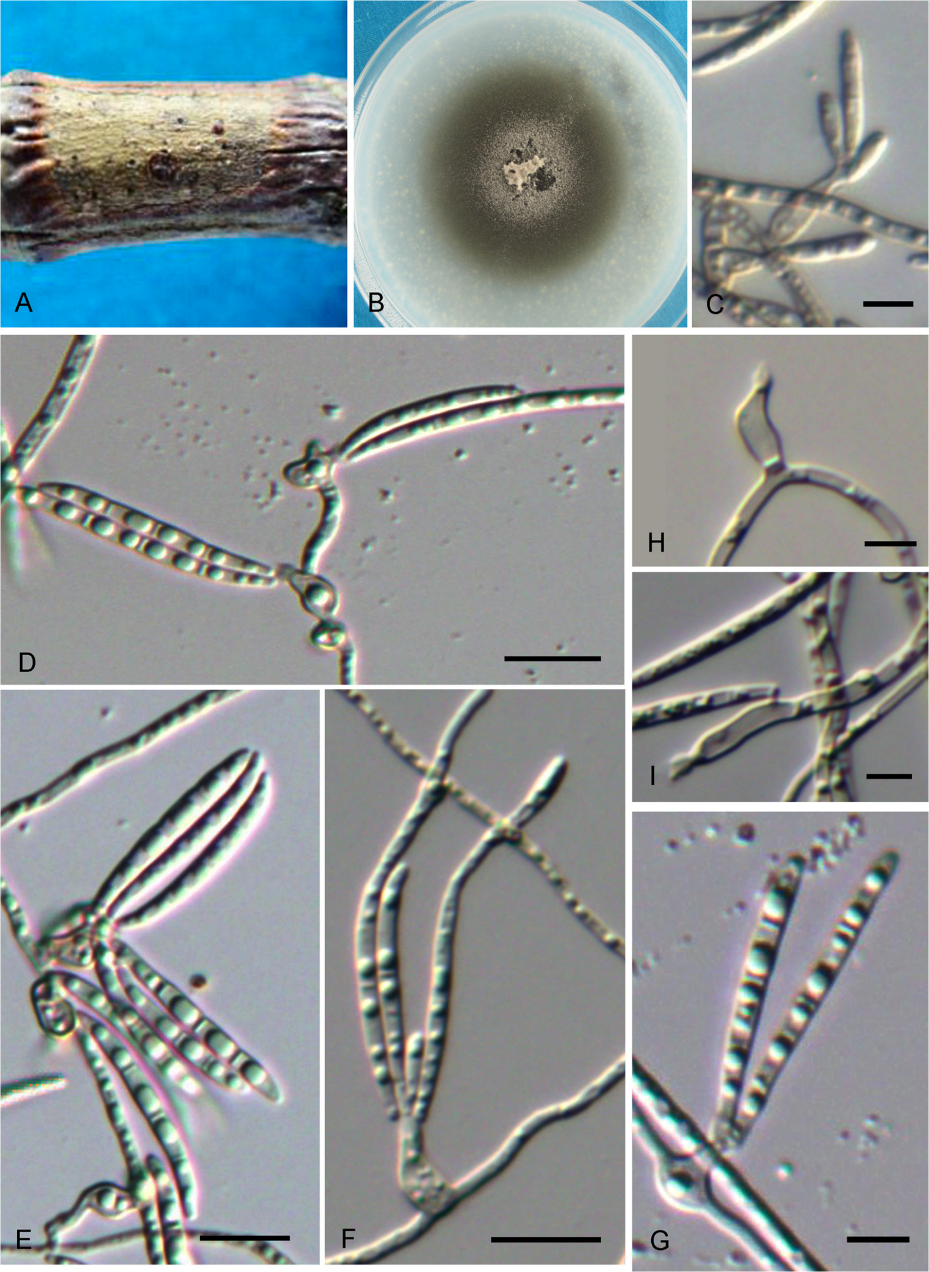
*Cyphellophora artocarpi*. A. SBFS signs occurring on twig of jackfruit. B, Colony on OA after 4 wk. C–G, Conidia and phialidic conidiogenous cells with collarettes. H–I, Conidiogenous cells. Bar = 5 μm.

***Cyphellophora artocarpi*.** G. Y. Sun & Liu Gao, sp. nov. (Fig 6)

MycoBank MB811436.

Hyphae thin- to slightly thick-walled, hyaline to pale grayish, smooth, septate, ramose, aerial and immersed, 1–2 μm wide. Conidiophores inconspicuous or reduced to a supporting cell basal to the conidiogenous cells. Conidiogenesis enteroblastic, phialidic. Phialides discrete, cylindrical to flask-shaped, wide in the middle, straight or gently curved, arising at right angles from undifferentiated hyphae, occasionally terminal, rarely intercalary, (4.5–)7–9.5(–13) × 2.5–3.5(–4) μm, with conspicuous funnel-shaped collarette, 1.5–2.5 μm diam, sometimes lighter than the body of phialides. Conidia hyaline, smooth, nearly straight to more commonly falcate or slightly sigmoid, with slightly truncate basis and rounded apices, 0–3-septate, (23.5–)28.5–35(–40) × 1.5–2 μm, aggregating in bundles. Chlamydospores and sclerotium absent.

Colonies on OA medium fast growing, attaining a diameter of 51 mm after 4 weeks at 25 C. Centre pale grey-brown, covered with loose, cottony mycelium and spreading with sparse aerial mycelium. Margin flattened, regular, greyish olive-green. Reverse pale brown.

*Etymology*: Named after the host from which it was collected.

*Holotype*: CHINA. HAINAN: Haikou. From twig of *Artocarpus heterophyllus* Lam., Oct 2010, C. Chen & H. C. Chen, CHCJHBJBLM (Holotype HMAS245770, ex-type culture CGMCC3.17496).

*Notes*: *C*. *artocarpi* is somewhat similar to *C*. *guyanensis* [5]. Phialides of both are discrete, ampulliform to flask-shaped and they both have long conidia. However, *C*. *guyanensis* exhibits more septa (3–6 septa) than *C*. *artocarpi* (0–3 septa). Moreover, the conidia of *C*. *artocarpi* are longer than those of *C*. *guyanensis*.

**Fig 7:**
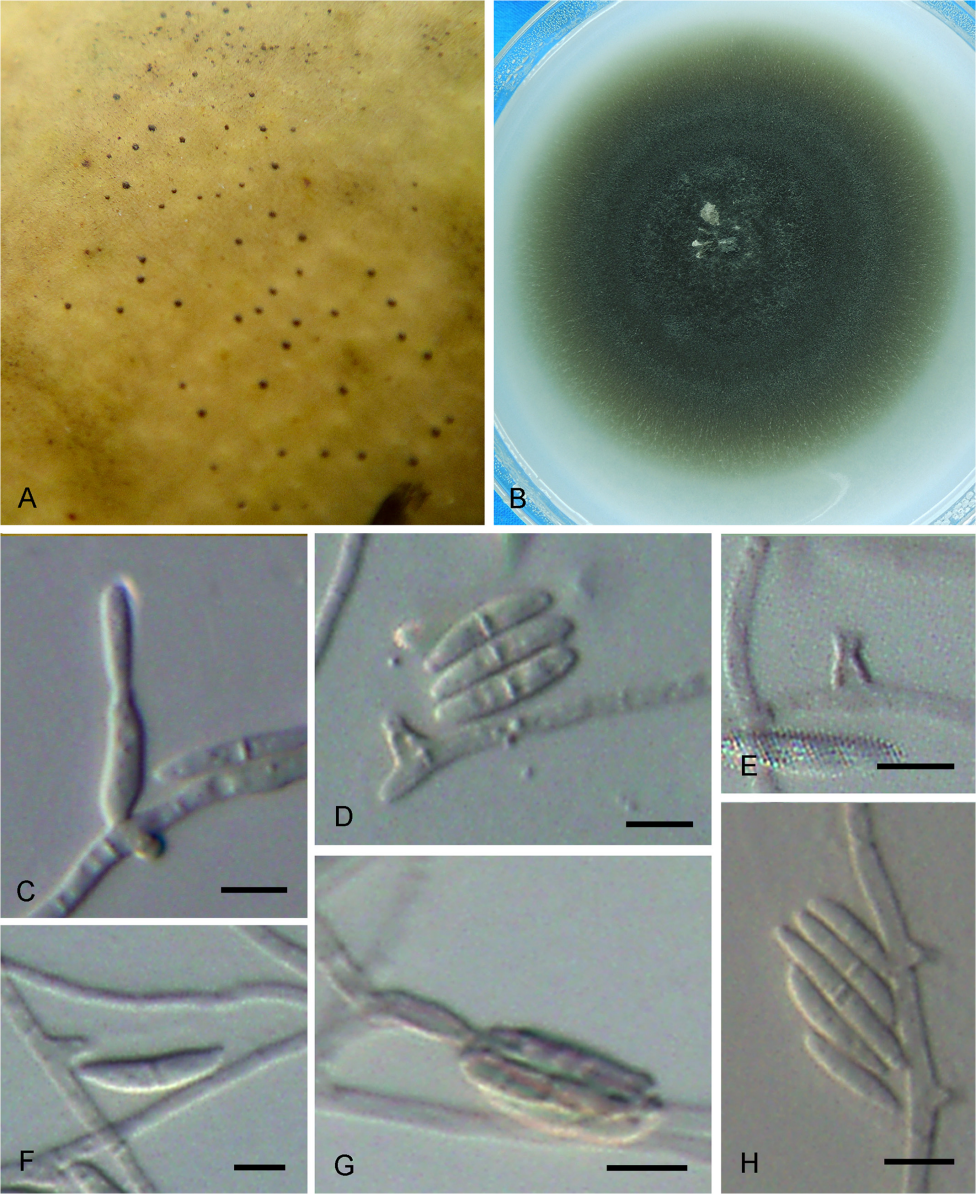
*Cyphellophora musae*. A. SBFS signs on the surface of Japanese banana fruit. B. Colony on OA after 4 wk. C–G. Conidia and phialidic conidiogenous cells with collarettes. H. Intercalary conidiogenous cells with sessile collarettes. Bar = 5 μm.

***Cyphellophora musae*.** G. Y. Sun & Liu Gao, sp. nov. (Fig 7)

MycoBank MB811438.

Hyphae thin- to slightly thick-walled, hyaline to pale grayish, smooth, septate, ramose, aerial and immersed, 1.5–2 μm wide. Conidiophores inconspicuous or reduced to a supporting cell basal to the conidiogenous cells. Conidiogenesis enteroblastic, phialidic. Phialides discrete, lateral and randomly distributed on the hyphae, wart-like to flask-shaped, arising at right angles from undifferentiated hyphae, sometimes intercalary, rarely terminal, 1–2.5 × 1–2(–6) μm, with conspicuous funnel-shaped collarette, 1–1.5 μm diam, sometimes darker than the body of phialides. Conidia hyaline, smooth, nearly straight to more commonly falcate or slightly sigmoid, with slightly truncate basis and rounded apices, 0–1-septate, (7–)9–15 × 1.5–2 μm, sometimes aggregating in bundles. Chlamydospores and sclerotium absent.

Colonies on OA medium relatively fast growing, attaining a diameter of 40 mm after 4 weeks at 25 C. Olivaceous grey in the center with sparse mycelium. Margin flattened, regular, greyish olive-green. Reverse pale brown.

*Etymology*: Named after the host from which it was collected.

*Holotype*: CHINA. GUANGDONG: Zhanjiang. From fruit of *Musa basjoo* Sieb. & Zncc., Oct 2011, L. Gao, W. H. Li & H. C. Chen, GLZJXJ41 (Holotype HMAS245771, ex-type culture CGMCC3.17497).

*Additional specimens examined*. CHINA. GUANGDONG: Guangzhou. From fruit of *Musa basjoo* Sieb. & Zncc., Oct 2011, L. Gao, W. H. Li & H. C. Chen, GLGZXJ9; CHINA. GUANGDONG: Maoming. From twig of *Sinobambusa tootsik* (Sieb.) Makino, Oct 2011, L. Gao, W. H. Li & H. C. Chen, GLMMZZ2.

*Notes*: This species is distinctive as both its phialides and conidia are smaller than those of closely related species. Many phialides of *C*. *musae* are like those of *C*. *pauciseptata* [6], which are intercalary with collarettes sessile on undifferentiated hyphae. However, the conidia of *C*. *musae* are much smaller than those of *C*. *pauciseptata* and the former have only 0–1 septum instead of several septa as described for *C*. *pauciseptata*.

**Fig 8:**
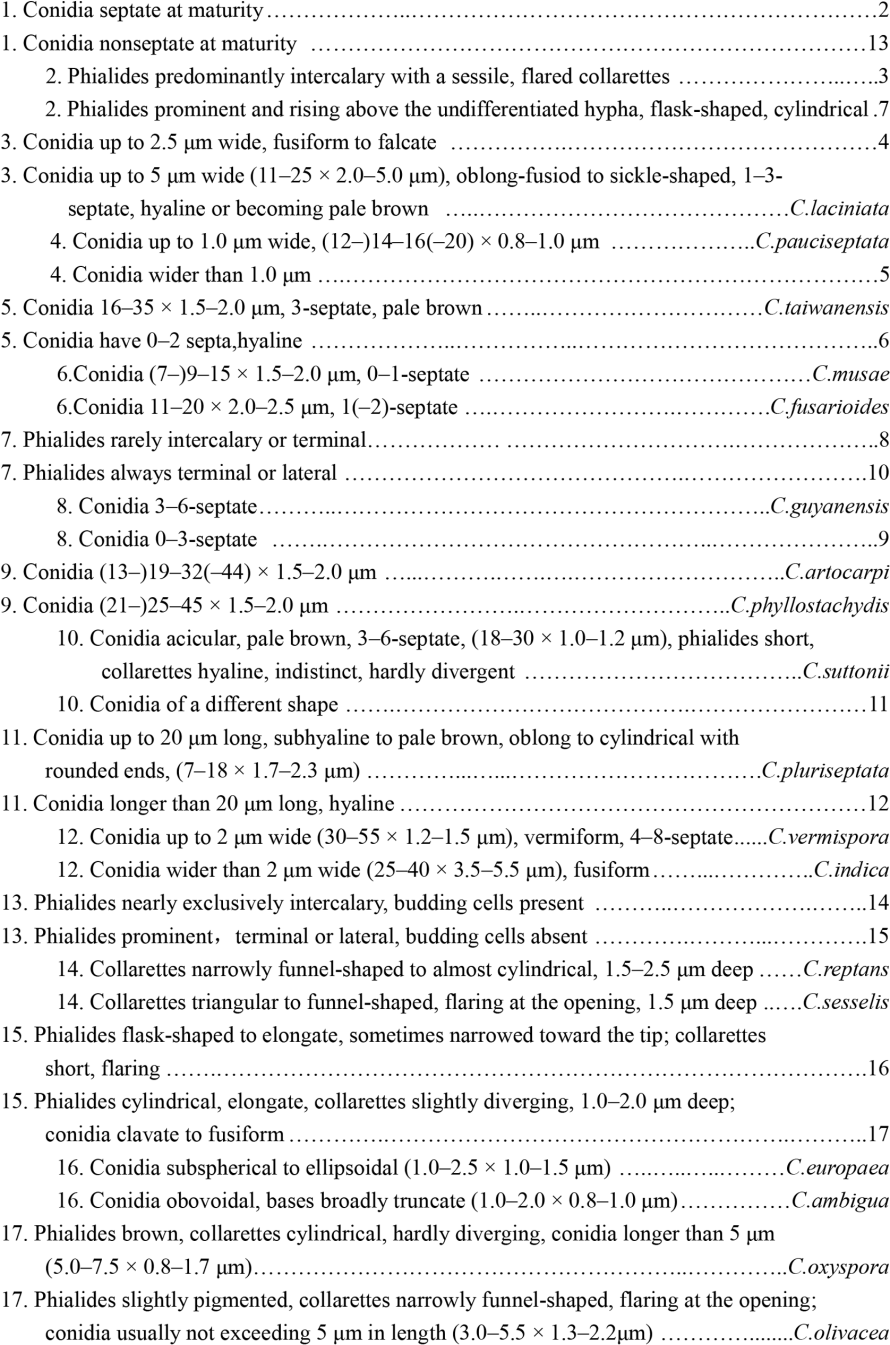
Key to species accepted in *Cyphellophora*.

Different species of *Cyphellophora* can be distinguished by the conidia size, septum number, phialide shape, size, position and collarette shape. Based on the key established by Réblová et al. [9], a new key to species of *Cyphellophora* was set up as in Fig 8.

### Pathogenicity test

**Fig 9:**
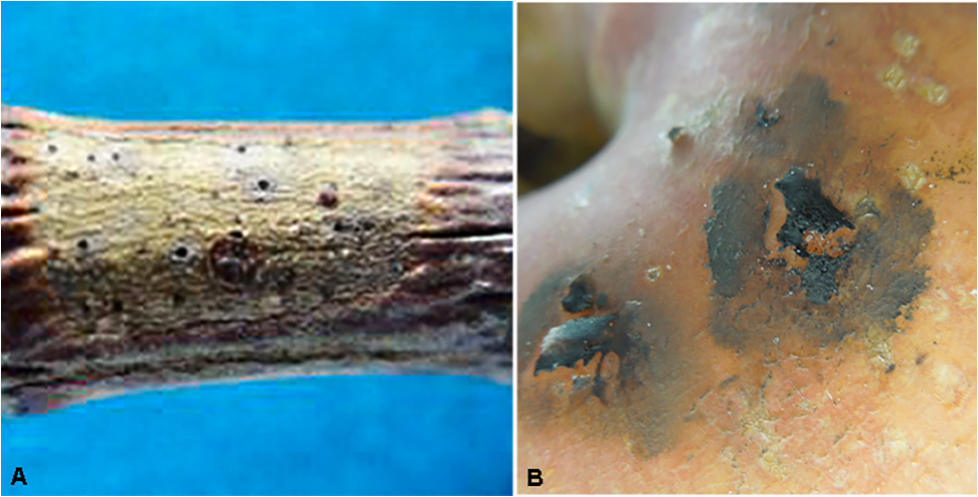
Colonies of *Cyphellophora artocarpi* produced on jackfruit (original host) and on apple (pathogenicity test). A. On jackfruit. B. On apple.

The representative isolates of the three new species of *Cyphellophora* were selected for pathogenicity tests on apple fruit. After 6 wk of inoculation in the incubator, *C*. *artocarpi* CHCJHBJBLM developed characteristic SBFS signs on apple. However, the colony morphology exhibited on apple was different from that on the original host. The original colonies presented a ramose (RS) mycelial type [21] containing both sclerotium-like bodies and mycelial mats on the twig of *Artocarpus heterophyllus*, whereas it exhibited a fuliginous (FG) mycelial type after laboratory inoculation, producing only mycelial mats (Fig 9) on the apple surface. After re-isolating the sclerotium-like bodies and re-sequencing the isolates, their ITS sequences were the same as the original ones. The control apple fruit did not show any signs. This result indicated that apple can be a potential host of *C*. *artocarpi*, and therefore that this species could be of economic importance as an apple pathogen. The isolates of *C*. *phyllostachydis* and *C*. *musae* did not produce any colonies on apple.

## Discussion

Formerly, the genus *Cyphellophora* G. A. de Vries was a polyphyletic group within the order Chaetothyriales [6, 9, 48–51]. *Cyphellophora laciniata* G. A. de Vries, the type species of *Cyphellophora*, and other members of this genus clustered together with species recognized as *Phialophora* Medlar, whereas *C*. *eugeniae* and *C*. *hylomeconis* grouped with *Exophiala eucalyptorum* and *Vonarxia vagans* [9]. Réblová et al. amended *Cyphellophora* to accommodate 15 species in a novel evolutionary family Cyphellophoraceae within the Chaetothyriales, and transferred *Cyphellophora eugeniae* and *C*. *hylomeconis* to two novel lineages as *Aphanophora eugeniae* and *Camptophora hylomeconis*, respectively, in the family Chaetothyriaceae (Chaetothyriales) [9]. The genus *Cyphellophora* became a monophyletic lineage. They redefined the concept of *Cyphellophora* as having melanized thalli with phialidic conidiogenesis, phialidic openings inserted directly on hyphae or occasionally on flask-shaped conidiogenous cells, and producing small clusters of olivaceous, septate or non-septate, mostly curved conidia. Based on the alignment of the ITS and ITS-LSU-TUB2-RPB1 matrix genes, our three new species, *Cyphellophora phyllostachysdis*, *C*. *artocarpi* and *C*. *musae*, were situated in *Cyphellophora* G.A. de Vries emend. Réblová & Unter supported by high bootstrap values. The morphological characters also clearly supported the phylogenetic identification of *Cyphellophora*.

ITS1 and ITS2 regions play an important role in folding, modifying, assembling and splitting rRNA genes during the maturation process [52–54] and the secondary structures of ITS were highly conserved in family or higher taxonomic levels [9, 55–59]. Coleman also compared the difference of conserved sequence on ITS1 of *Gonium* and *Astrephomene* [55] and ITS2 among different eukaryotes [59], indicating changes of 2D structure among different orders, classes, etc. Réblová et al. detecting the difference of three members of Chaetothyriales—Cyphellophoraceae (the family containing *Cyphellophora*), Herpotrichiellaceae (containing *Phialophora*) and Chaetothyriaceae—by comparing 2D structures of ITS1 and ITS2 and three evolutionary motifs were observed within ITS2. The 2D structure of *C*. *phyllostachysdis*, *C*. *artocarpi* and *C*. *musae* also supports the taxonomic status determined by phylogenetic and morphological analysis. Most of the 2D structure characters of the ITS1 and ITS2 were consistent with those of Cyphellophoraceae [9], including the purine-purine pair on H3 helix of ITS1 and three evolutionary motifs on ITS2. These three species also had their own distinct 2D characters. For example, in *C*. *phyllostachysdis*, the bottom base pair of the H2 hairpin loop of ITS1 was U-A, whereas in other species of *Cyphellophora* (except *C*. *sessilis*) this base pair is C = G (Fig 3C). Furthermore, the T2 hairpin of ITS2 reached to 12 nt in *C*. *phyllostachysdis* (Fig 4C) which is the longest of any *Cyphellophora* species. For *C*. *artocarpi*, the H5 duplex of ITS2 only had 3 bp whereas other species (except *C*. *guyanensis*) have 4 bp.

The genus *Cyphellophora* colonizes a variety of habitats including plant, animal and abiotic substrates. *Cyphellophora europaea*, *C*. *ambigua*, *C*. *europaea*, *C*. *fusarioides*, *C*. *laciniata*, *C*. *pauciseptata*, *C*. *pluriseptata*, *C*. *suttonii* and *C*. *oxyspora* were reported from human or animal materials, such as nails, superficial skin, foot and toe [1, 2, 5, 6, 9, 10, 12, 60, 61]. *C*. *europaea* can cause hyperkeratosis of humans and affect the skin of diabetic patients [10, 62]. This species grows on the surface of human and animal skin and causes superficial infections under appropriate conditions [50]. Some species can be isolated from decaying plant materials and are believed to be saprophytes. For instance, a strain of *C*. *guyanensis* was isolated from rotting wood [6] and isolates of *C*. *oxyspora* from decaying leaves [12]. *C*. *vermispora*, *C*. *indica* and *C*. *taiwanensis* were isolated from living plant materials such as stems, roots, and leaves [2, 4, 5, 8, 11, 12]. Other substrates from a range of environments are common for *Cyphellophora*. For example, strains of *C*. *reptans* were isolated from food and drinking water [11], *C*. *suttonii* was detected in soil [50] and strains of *C*. *sessilis* were recovered from resin and marble [11]. The species *C*. *sessilis* [16] and the three new species of *Cyphellophora* described in this study colonize the surface of plants and SBFS disease as epiphytic fungi. The phylogenetic relationship between these different species can be quite close. For instance, *C*. *europaea* and *C*. *phyllostachysdis* have a very close relationship according to phylogeny analysis (Figs 1 and 2). *C*. *europaea* isolates are found in association with human or mammal samples and can cause hyperkeratosis [10] or asymptomatic colonization [62] on humans whereas *C*. *phyllostachysdis* was isolated from twigs of bamboo displaying SBFS signs. The virulence of *C*. *phyllostachysdis* on humans has not been assessed.

Pathogenicity tests on apple fruit in our study indicated that the isolate of *C*. *artocarpi* from jackfruit (*Artocarpus heterophyllus*) could cause SBFS on apple. Grabowski [16] formerly reported that *C*. *sessilis* (= *Phialophora sessilis*) was one of the SBFS pathogens on apple in southern Poland. This information further broadens understanding of the diversity of fungi in the SBFS complex, and could be useful to plant pathologists and fruit growers in improving management of this economically important disease.

Batzer et al. [21] classified mycelial types of SBFS fungi as ramose, punctate, ridged honeycomb, fuliginous, flyspeck, compact speck and discrete speck, and available evidence indicated that each species is characterized by a single mycelial type. Interestingly, in this study we found the same species exhibited two distinct mycelial types on different hosts: *Cyphellophora artocarpi* produced fuliginous colonies on apple, whereas it produced the compact speck mycelial type on jackfruit. This is the first evidence of plasticity of mycelial type by a single SBFS species on different plant hosts. However, it should be regarded as preliminary because i) *C*. *artocarpi* has not been found on apple under field conditions and ii) the same isolates of *C*. *artocarpi* should be inoculated onto both hosts in the field to confirm that the distinct mycelial types develop there as observed under controlled conditions.

## Supporting Information

S1 Table Cultures of *Cyphellophora* spp. used for morphological and molecular studies in species of *Cyphellophora*. (DOCX)
