# Supplementary material for: Three New Species of Cyphellophora (Chaetothyriales) Associated with Sooty Blotch and Flyspeck
Source: PLoS One. 2015 Sep 23;10(9):e0136857. doi: 10.1371/journal.pone.0136857 (PMC4580582; doi:10.1371/journal.pone.0136857)
Supplement: S1 Table — (DOCX) [file pone.0136857.s001.docx]

**S1 Table. Cultures of *Cyphellophora* spp. used for morphological and molecular studies in species of *Cyphellophora*.**

| Species | Strain numbers | Source | Locality | GenBank accession numbers | | | |
| --- | --- | --- | --- | --- | --- | --- | --- |
|  |  |  |  | ITS^b^ | LSU^c^ | TUB2^d^ | RPB1^e^ |
| *C. ambigua* | CBS^a^ 235.93 (T) | Human, toenail | Netherland | JQ766431 | JQ766480 | JQ766340 | JQ766386 |
| *C. europaea* | CBS 101466 (T) | Human, foot | Netherland | JQ766443 | JQ766491 | JQ766365 | JQ766395 |
|  | CBS 218.78 | Human, nail | Netherland | JQ766441 | JQ766488 | JQ766366 | JQ766393 |
|  | CBS 129.96 | Human, foot skin | Germany | JQ766440 | JQ766487 | JQ766364 | JQ766392 |
| *C. guyanensis* | CBS 124764 (T) | leaves of *Eucalyptus* sp. | Australia | JQ766432 | JQ766481 | JQ766337 | JQ766387 |
|  | CBS 126014 | Tucum palm | Brazil | JQ766434 | JQ766483 | JQ766339 | JQ766389 |
|  | CBS 126020 | Rotting wood | Brazil | JQ766435 | JQ766484 | JQ766341 | JQ766390 |
|  | CBS 125756 | Roof of patient’s house | Brazil | JQ766433 | JQ766482 | JQ766338 | JQ766388 |
| *C. laciniata* | CBS 190.61 (T) | Human, skin | Switzerland | JQ766423 | JQ766472 | JQ766329 | JQ766378 |
|  | CBS 174.79 | Human, foot skin | Netherland | JQ766422 | JQ766471 | JQ766328 | JQ766377 |
| *C. olivacea* | CBS 122.74(T) | Moist wall paper | Germany | KC455247 | KC455260 | KC455230 | — |
|  | CBS 123.74 | stem of *Chamaerops humil* | Netherland | KC455248 | KC455261 | KC455231 | — |
| *C. oxyspora* | CBS 698.73 (T) | Decaying leaf | Sri Lanka | JQ766450 | JQ766498 | JQ766375 | JQ766402 |
|  | CBS 416.89 | Human, skin | Denmark | JQ766449 | JQ766497 | JQ766374 | JQ766401 |
| *C. pauciseptata* | CBS 284.85 (T) | Human, hand skin | Netherland | JQ766466 | JQ766515 | JQ766358 | JQ766415 |
| *C. pluriseptata* | CBS 286.85 (T) | Human, toenail | Netherland | JQ766429 | JQ766478 | JQ766335 | JQ766384 |
|  | CBS 109633 | Human, skin | Germany | JQ766430 | JQ766479 | JQ766336 | JQ766385 |
| *C. reptans* | CBS 113.85 (T) | Foodstuffs | Sweden | JQ766445 | JQ766493 | JQ766370 | JQ766397 |
|  | CBS 152.90 | Human, nail | Netherland | JQ766446 | JQ766494 | JQ766371 | JQ766398 |
|  | CBS 458.92 | Human, skin | Netherland | JQ766447 | JQ766495 | JQ766372 | JQ766399 |
|  | CBS 120903 | Drinking water | Germany | JQ766448 | JQ766496 | JQ766373 | JQ766400 |
| *C. sessilis* | CBS 243.85 (T) | Resin of *Picea abies* | Netherland | EU514700 | EU514700 | KC455234 | — |
|  | CBS 238.93 | Styrene | Netherland | AY857541 | KC455264 | KC455235 | — |
| *C. suttonii* | CBS 449.91 (T) | Dog, internal ear | U.S.A. | JQ766459 | JQ766500 | JQ766342 | JQ766404 |
| *C. fusarioides* | CBS 130291 (T) | Man, bronchial lavage fluid | Israel | JQ766439 | JQ766486 | JQ766363 | JQ766391 |
| *C. vermispora* | CBS 228.86 (T) | root of *Triticum aestivum* | Germany | KC455244 | KC455257 | KC455227 | JQ766381 |
|  | CBS 227.86 | root of *Hordeum vulgare* | Germany | JQ766425 | JQ766474 | JQ766331 | JQ766380 |
|  | CBS 122852 | man skin, foot | Netherland | JQ766427 | JQ766476 | JQ766333 | JQ766382 |
| *C. phyllostachysdis* | HLHNZWYZZ08 | twigs of bamboo | China | KP010371 | KP122933 | KP122929 | KP122924 |
| *C. artocarpi* | CHCJHBJBLM | twigs of jackfruit | China | KP010367 | KP122930 | KP122925 | KP122920 |
| *C. musae* | GLZJXJ41 | fruit of Japanese banana | China | KP010370 | KP122932 | KP122927 | KP122922 |
|  | GLMMZZ4 | twigs of bamboo | China | KP010369 | KP122934 | KP122928 | KP122921 |
|  | GLGZXJ9B | fruit of Japanese banana | China | KP010368 | KP122931 | KP122926 | KP122923 |
| *Cladophialophora immunda* | CBS 834.96 |  |  | EU137318 | KC809990 | EU137203 | — |

^a^CBS: CBS-KNAW Fungal Biodiversity Centre, Utrecht, The Netherlands

^b^ITS: internal transcribed spacers 1 and 2 together with 5.8S nrDNA

^c^LSU: nuclear large subunit rDNA gene

^d^TUB2: partial β-tubulin (tub2)

^e^RPB1: DNA dependent RNA polymerase II largest subunit
